# Supplementary material for: Durability of Antibody Response after Primary Pneumococcal Double-Dose Prime-Boost Vaccination in Adult Kidney Transplant Recipients and Candidates: 18-Month Follow-Up in a Non-Blinded, Randomised Clinical Trial
Source: Vaccines (Basel). 2022 Jul 7;10(7):1091. doi: 10.3390/vaccines10071091 (PMC9323946; doi:10.3390/vaccines10071091)
Supplement: Supplementary file 1 [file vaccines-10-01091-s001.zip › vaccines-1783684-SI.pdf]

Supplementary Figure S1: Geometric mean concentrations for 12 pneumococcal serotype-specific IgG antibodies in mg/L at baseline, week 12 (pre-PPV23), week 17, and week 96. Note that the scale of the y-axis is different for all serotypes.

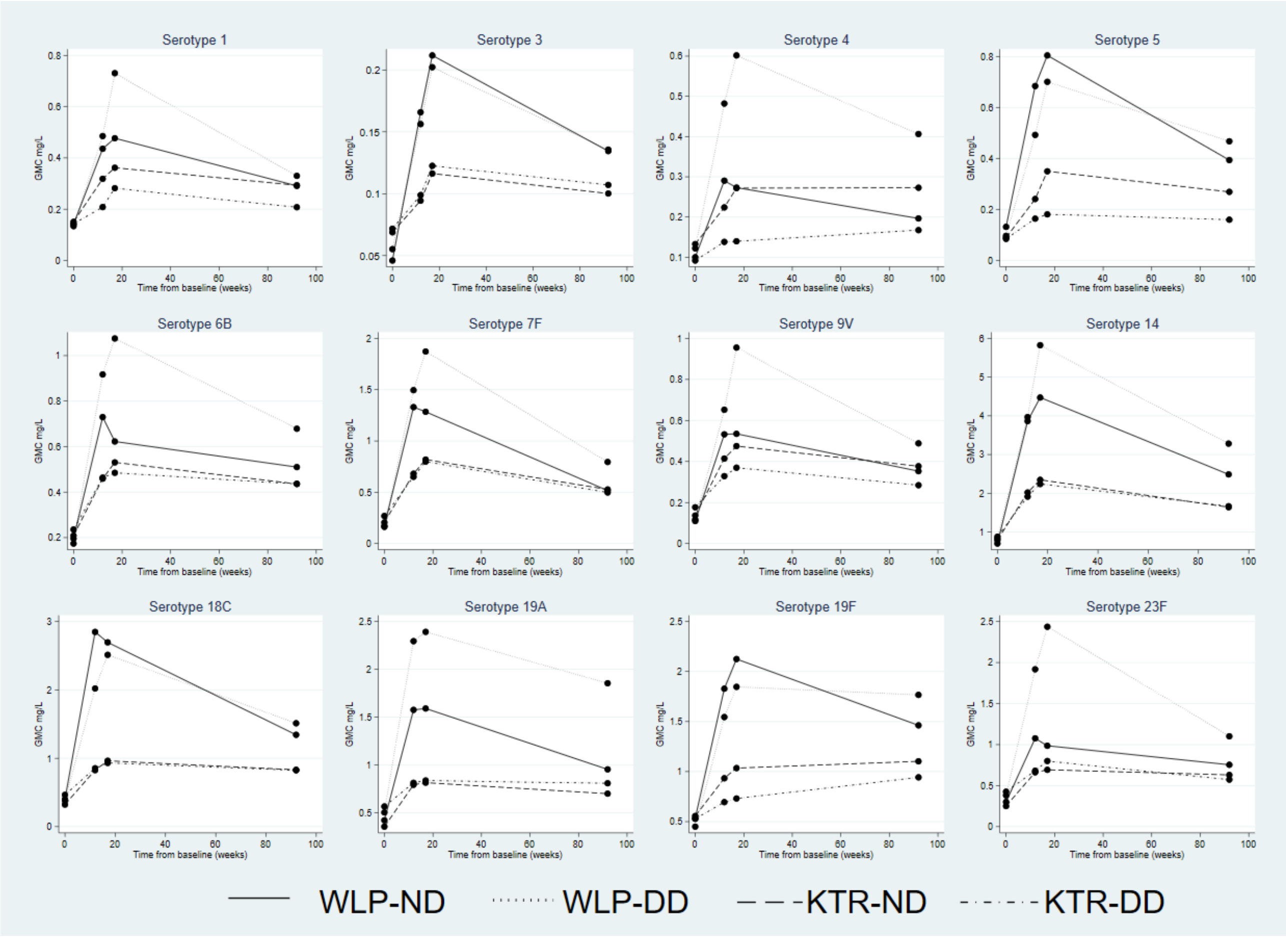

Abbreviations: GMC, Geometric mean concentration; WLP-ND, patients on the kidney transplant waiting list – normal dose; WLP-DD, patients on the kidney transplant waiting list – double dose; KTR-ND, kidney transplant recipient – normal dose; KTR-DD, kidney transplant recipient – double dose; PPV23, 23-valent pneumococcal polysaccharide.

Supplementary Table S1: Geometric mean concentrations (95% confidence interval) for 12 pneumococcal serotype-specific IgG antibodies at each visit, with p-values for comparisons within groups and between normal and double dosages.

Serotype	Treatment groups	Baseline GMC (95%CI)	P-value Baseline: Comp. to DD	Week12 GMC (95%CI)	P-value Week 12: Comp. to DD	P-value Week 12: Comp. to Baseline	Week 17 GMC (95%CI)	P-value Week 17: Comp. to DD	P-value Week 17: Comp. to Week 12	P-value Week 17: Comp. to Baseline	Week 96 GMC (95%CI)	P-value Week 96: Comp. to DD	P-value Week 96: Comp. to Baseline
**1**	WLP-ND	0.14 (0.09-0.21)	0.918	0.44 (0.22-0.86)	0.487	≤0.001	0.48 (0.25-0.91)	0.176	0.781	≤0.001	0.29 (0.16-0.55)	0.605	≤0.001
	WLP-DD	0.13 (0.09-0.20)		0.49 (0.27-0.86)		≤0.001	0.73 (0.41-1.30)		≤0.001	≤0.001	0.33 (0.20-0.55)		≤0.001
	KTR-ND	0.15 (0.10-0.22)	0.506	0.32 (0.19-0.54)	0.232	≤0.001	0.36 (0.22-0.60)	0.434	0.264	≤0.001	0.29 (0.18-0.47)	0.264	≤0.001
	KTR-DD	0.14 (0.09-0.22)		0.21 (0.13-0.33)		≤0.001	0.28 (0.16-0.48)		0.033	0.001	0.21 (0.14-0.32)		0.046
**3**	WLP-ND	0.05 (0.03-0.07)	0.690	0.17 (0.09-0.31)	0.906	≤0.001	0.21 (0.11-0.41)	0.963	0.245	≤0.001	0.13 (0.08-0.23)	0.842	≤0.001
	WLP-DD	0.06 (0.03-0.09)		0.16 (0.08-0.29)		≤0.001	0.20 (0.11-0.38)		0.122	≤0.001	0.14 (0.08-0.22)		≤0.001
	KTR-ND	0.07 (0.05-0.10)	0.685	0.09 (0.06-0.14)	0.702	0.014	0.12 (0.07-0.18)	0.764	0.035	≤0.001	0.10 (0.07-0.15)	0.698	0.005
	KTR-DD	0.07 (0.04-0.12)		0.10 (0.06-0.18)		≤0.001	0.12 (0.07-0.22)		0.008	≤0.001	0.11 (0.06-0.19)		0.055
**4**	WLP-ND	0.10 (0.07-0.14)	0.595	0.29 (0.16-0.51)	0.204	≤0.001	0.27 (0.15-0.49)	0.079	0.658	≤0.001	0.20 (0.12-0.31)	0.026	≤0.001
	WLP-DD	0.12 (0.08-0.20)		0.48 (0.24-.96)		≤0.001	0.60 (0.29-1.23)		.236	≤0.001	0.41 (0.26-0.64)		≤0.001
	KTR-ND	0.13 (0.09-0.19)	0.198	0.22 (0.14-0.36)	0.153	≤0.001	0.27 (0.17-0.43)	0.028	0.048	≤0.001	0.27 (0.19-0.40)	0.055	≤0.001
	KTR-DD	0.09 (0.07-0.12)		0.14 (0.10-0.19)		0.006	0.14 (0.10-0.20)		0.936	0.027	0.17 (0.12-0.23)		0.003
**5**	WLP-ND	0.13 (0.06-0.28)	0.836	0.68 (0.30-1.57)	0.647	≤0.001	0.80 (0.38-1.70)	0.840	0.644	≤0.001	0.39 (0.19-0.82)	0.729	≤0.001
	WLP-DD	0.10 (0.06-0.16)		0.49 (0.22-1.10)		≤0.001	0.70 (0.32-1.53)		0.616	≤0.001	0.47 (0.23-0.94)		≤0.001
	KTR-ND	0.09 (0.06-0.14)	0.782	0.24 (0.13-0.45)	0.552	≤0.001	0.35 (0.19-0.64)	0.096	0.005	≤0.001	0.27 (0.15-0.48)	0.187	≤0.001
	KTR-DD	0.08 (0.05-0.14)		0.16 (0.10-0.28)		0.004	0.18 (0.11-0.30)		0.893	0.003	0.16 (0.09-0.27)		≤0.001
**6B**	WLP-ND	0.20 (0.12-0.32)	0.767	0.73 (0.32-1.67)	0.636	≤0.001	0.62 (0.27-1.42)	0.359	0.072	≤0.001	0.51 (0.26-1.01)	0.487	≤0.001
	WLP-DD	0.17 (0.11-0.28)		0.92 (0.42-1.99)		≤0.001	1.08 (0.52-2.22)		0.452	≤0.001	0.68 (0.36-1.27)		≤0.001
	KTR-ND	0.21 (0.14-0.31)	0.995	0.46 (0.25-0.84)	0.902	≤0.001	0.53 (0.30-0.94)	0.693	0.230	≤0.001	0.44 (0.26-0.73)	0.737	≤0.001
	KTR-DD	0.24 (0.14-0.40)		0.46 (0.23-0.94)		≤0.001	0.49 (0.25-0.93)		0.177	0.002	0.44 (0.24-0.80)		0.002
**7F**	WLP-ND	0.17 (0.10-0.30)	0.890	1.33 (0.71-2.48)	0.636	≤0.001	1.29 (0.69-2.41)	0.367	0.349	≤0.001	0.52 (0.30-0.90)	0.303	0.001
	WLP-DD	0.16 (0.09-0.29)		1.50 (0.73-3.05)		≤0.001	1.87 (0.96-3.65)		0.295	≤0.001	0.80 (0.42-1.52)		≤0.001
	KTR-ND	0.21 (0.14-0.30)	0.353	0.68 (0.38-1.21)	0.809	≤0.001	0.82 (0.47-1.44)	0.883	0.177	≤0.001	0.53 (0.32-0.87)	0.861	≤0.001
	KTR-DD	0.27 (0.17-0.42)		0.65 (0.42-1.02)		≤0.001	0.80 (0.50-1.27)		0.272	≤0.001	0.50 (0.33-0.75)		≤0.001
**9V**	WLP-ND	0.11 (0.07-0.17)	0.844	0.53 (0.29-0.97)	0.595	≤0.001	0.54 (0.31-.92)	0.171	0.245	≤0.001	0.35 (0.22-0.58)	0.377	≤0.001
	WLP-DD	0.12 (0.08-0.17)		0.65 (0.34-1.26)		≤0.001	0.96 (0.55-1.67)		0.982	≤0.001	0.49 (0.28-0.85)		≤0.001
	KTR-ND	0.14 (0.09-0.20)	0.400	0.41 (0.22-0.78)	0.601	≤0.001	0.48 (0.26-0.88)	0.510	0.331	≤0.001	0.38 (0.22-0.63)	0.441	≤0.001
	KTR-DD	0.18 (0.11-0.29)		0.33 (0.19-0.56)		≤0.001	0.37 (0.22-0.62)		0.288	≤0.001	0.28 (0.17-0.48)		0.161

**14**	WLP-ND	0.70 (0.34-1.46)	0.722	3.86 (2.07-7.23)	0.802	≤0.001	4.47 (2.25-8.89)	0.630	0.221	≤0.001	2.49 (1.24-4.97)	0.565	≤0.001
	WLP-DD	0.83 (0.44-1.57)		3.97 (1.99-7.90)		≤0.001	5.83 (2.94-11.54)		0.023	≤0.001	3.28 (1.64-6.57)		≤0.001
	KTR-ND	0.79 (0.47-1.33)	0.792	2.02 (1.15-3.53)	0.958	≤0.001	2.35 (1.31-4.21)	0.930	0.172	≤0.001	1.64 (0.95-2.84)	0.966	0.002
	KTR-DD	0.88 (0.48-1.58)		1.91 (1.10-3.33)		≤0.001	2.24 (1.26-3.97)		0.053	≤0.001	1.66 (0.99-2.79)		0.017
**18C**	WLP-ND	0.40 (0.25-0.63)	0.889	2.85 (1.68-4.82)	0.824	≤0.001	2.70 (1.51-4.82)	0.803	0.453	≤0.001	1.34 (0.81-2.24)	0.734	≤0.001
	WLP-DD	0.38 (0.24-0.62)		2.02 (1.07-3.80)		≤0.001	2.51 (1.28-4.93)		0.802	≤0.001	1.51 (0.88-2.60)		≤0.001
	KTR-ND	0.32 (0.22-0.47)	0.200	0.83 (0.51-1.35)	0.883	≤0.001	0.96 (0.57-1.62)	0.819	0.006	≤0.001	0.83 (0.54-1.29)	0.755	≤0.001
	KTR-DD	0.47 (0.30-0.72)		.85 (0.56-1.31)		≤0.001	0.93 (0.56-1.54)		0.029	≤0.001	0.82 (0.51-1.33)		0.008
**19A**	WLP-ND	0.42 (0.22-0.79)	0.668	1.58 (0.73-3.41)	0.460	≤0.001	1.59 (0.70-3.61)	0.455	0.349	≤0.001	0.95 (0.44-2.05)	0.193	≤0.001
	WLP-DD	0.50 (0.25-1.01)		2.29 (1.12-4.69)		≤0.001	2.39 (1.09-5.27)		0.236	≤0.001	1.85 (0.93-3.68)		≤0.001
	KTR-ND	0.35 (0.21-0.60)	0.282	.79 (0.41-1.51)	0.995	0.006	0.81 (0.41-1.62)	0.911	0.474	≤0.001	0.70 (0.38-1.30)	0.646	≤0.001
	KTR-DD	0.56 (0.32-0.99)		.81 (0.48-1.37)		0.029	0.84 (0.46-1.53)		0.879	0.091	0.81 (0.50-1.31)		0.075
**19F**	WLP-ND	0.53 (0.32-0.88)	0.813	1.83 (0.99-3.39)	0.605	≤0.001	2.13 (1.17-3.86)	0.779	0.565	≤0.001	1.46 (0.86-2.48)	0.501	≤0.001
	WLP-DD	0.45 (0.29-0.69)		1.54 (0.79-3.04)		≤0.001	1.85 (0.96-3.55)		0.846	≤0.001	1.77 (1.07-2.92)		≤0.001
	KTR-ND	0.56 (0.36-0.86)	0.883	.93 (0.54-1.61)	0.568	≤0.001	1.04 (0.59-1.82)	0.387	0.216	≤0.001	1.10 (0.66-1.83)	0.823	≤0.001
	KTR-DD	0.53 (0.34-0.81)		.69 (0.44-1.10)		0.034	0.73 (0.44-1.21)		0.741	0.161	0.94 (0.59-1.50)		0.011
**23F**	WLP-ND	0.30 (0.20-0.45)	0.487	1.08 (0.59-1.97)	0.194	≤0.001	0.99 (0.52-1.89)	0.050	0.934	≤0.001	0.75 (0.43-1.34)	0.416	≤0.001
	WLP-DD	0.38 (0.25-0.57)		1.92 (0.99-3.73)		≤0.001	2.44 (1.26-4.71)		0.716	≤0.001	1.10 (0.57-2.11)		≤0.001
	KTR-ND	0.25 (0.18-0.35)	0.053	0.66 (0.39-1.13)	0.932	≤0.001	0.69 (0.39-1.24)	0.700	0.093	≤0.001	0.63 (0.38-1.04)	0.967	≤0.001
	KTR-DD	0.43 (0.29-0.63)		0.68 (0.45-1.02)		≤0.001	0.80 (0.51-1.26)		0.106	≤0.001	0.57 (0.38-0.87)		0.166

Abbreviations: GMC, Geometric mean concentration; CI, confidence interval; WLP-ND, patients on the kidney transplant waiting list – normal dose; WLP-DD, patients on the kidney transplant waiting list – double dose; KTR-ND, kidney transplant recipients – normal dose; KTR-DD, kidney transplant recipients – double dose; ND, normal dose; DD, double dose

Supplementary Table S2: T/B/NK cells at baseline for a subgroup of kidney transplant candidates and recipients.

	**WLPs** (n=32)	**KTRs** (n=58)	**p-value**
**Normal dosage vaccines, N (%)**	16 (50)	32 (55.2)	0.638
**Age, median years (IQR)**	50.5 (40-58)	50.5 (41-60)	0.406
**Absolute lymphocyte count x 10^3/µL, median (IQR)**	1.28 (1.06-1.65)	1.06 (0.77-1.58)	0.028
T-cells: CD3+ x10^3/µL, median (IQR	1.00 (0.73-1.28)	0.89 (0.58-1.25)	0.092
• *Helper T-cells: CD4+ x10^3/µL, median (IQR)*	0.66 (0.47-0.78)	0.53 (0.27-0.72)	0.046
• *Cytotoxic T-cells: CD8+ x10^3/µL, median (IQR)*	0.31 (0.19-0.54)	0.31 (0.2-0.43)	0.607
B-cells: CD19+ x10^3/µL, median (IQR)	0.08 (0.04-0.19)	0.07 (0.04-0.13)	0.357
NK-cells: CD3-/CD16-56+ x 10^3/µL, median (IQR)	0.18 (0.12-0.26)	0.12 (0.09-0.17)	0.007

Abbreviations: WLPs, patients on the kidney transplant waiting list; KTRs, kidney transplant recipients
